# Extracellular vesicle sizing and enumeration by nanoparticle tracking analysis

**DOI:** 10.3402/jev.v2i0.19671

**Published:** 2013-02-15

**Authors:** Chris Gardiner, Yannick J. Ferreira, Rebecca A. Dragovic, Christopher W.G. Redman, Ian L. Sargent

**Affiliations:** Nuffield Department of Obstetrics and Gynaecology, University of Oxford, Level 3, Women's Centre, John Radcliffe Hospital, Oxford, UK

**Keywords:** extracellular vesicles, nanoparticle tracking analysis, light scattering, standardisation

## Abstract

Nanoparticle tracking analysis (NTA) is a light-scattering technique that is useful for the rapid sizing and enumeration of extracellular vesicles (EVs). As a relatively new method, NTA has been criticised for a lack of standardisation. We propose the use of silica microspheres for the calibration of NTA measurements and describe in detail a protocol for the analysis of EVs by NTA which should minimise many of the sources of variability and imprecision associated with this technique.

Extracellular vesicles (EVs) play a key role in intercellular communication and are involved in many physiological and pathological conditions. Consequently, EVs have great potential as biomarkers in the diagnosis, prognosis and monitoring of disease. However, EV measurement presents unique challenges due to their exceptionally small size, low refractive index and polydispersity.

Nanoparticle tracking analysis (NTA) is a light-scattering method that is useful for the rapid assessment of EV size and concentration (1). During NTA measurement, particles (in this case, EVs) are illuminated by a focussed laser beam passed through particles in suspension. The light scattered by each individual particle in the field of view is focussed by the microscope onto the image sensor of the video camera. The NTA software identifies and tracks each particle, thus enabling measurement of the mean square displacement (MSD) of particle movement, which is used together with the temperature and the viscosity of the liquid containing the particle to calculate particle size through the Stokes-Einstein equation. A detailed explanation of the principle and performance of the technique and comparison with alternative methods are beyond the scope of this paper, but have been reported elsewhere (1–5).

There are 5 types of NTA instruments currently produced by Nanosight (Amesbury, UK): the basic LM10 is based on a conventional microscope and is fitted with a standard CCD camera; the LM10-HS is similar to the LM10 but has a more sensitive CMOS camera; the LM20 is a compact version of the LM10 with a CCD camera; the LM200 is identical to the LM20 but with a CMOS camera; and the NS500 is a larger instrument with automated sample introduction, sample handling, a computer-controlled motorised stage and a CCD or CMOS camera. Some older instruments were equipped with an EMCCD camera. Each instrument is supplied with a violet (405 nm), blue (488 nm), green (532 nm), or red (638 nm) laser and may be fitted with appropriate long pass or band pass filters to facilitate fluorescence measurements. An optional syringe pump for sample introduction may also be fitted to the LM10 and NS500 models. The NS500 may also be equipped to measure zeta-potential. NTA was first marketed in 2006, and application of NTA to the measurement of EVs is a relatively recent development (1). Consequently, there has been little work on standardisation, and a criticism of NTA has been the lack of agreement with other methods. Standardisation is necessary for any analytical method, to ensure comparability of methods performed at different times, by different operators, in different laboratories. It may be achieved through the use of written standards, reference methods and reference materials. In this document, we will describe standardised protocols used in our laboratory that we believe will be broadly applicable to others using this technique.

## Sources of variability

Size measurements obtained by NTA are generally accurate (within 5% of expected size) if the appropriate video capture and analysis settings are used (see the Methods section). Potential causes of error are inaccurate temperature measurement, incorrect assessment of viscosity and external vibration. Constant temperature monitoring with a correctly calibrated digital thermometer is necessary for each measurement (this is performed automatically in several models). The default viscosity setting in NTA is that of water and, as most samples are diluted several fold in buffered saline, this is generally appropriate. The most recent software release (NTA 2.3) displays a warning if vibration is suspected and can correct for small steady vibrations.

Potential sources of variability in NTA absolute concentration measurements include the type of camera, laser wavelength, depth of laser beam, cleanliness/wear of the metallised glass optical flat surface, duration of measurements, optical alignment, vibration and operator proficiency. These factors also affect the size resolution that varies between instruments and is also highly dependent on the refractive index of the particles being studied.

Consequently, video acquisition and analysis settings are not transferrable between instruments with different specifications. In order to overcome this inherent variability, we propose the use of the calibration procedure described in this paper. We have also detailed a series of measures that we have found useful in reducing variability in the technical comments section. Pre-analytical variables, which remain a major source of variability in part due to a lack of consensus, are beyond the scope of this paper, but have been covered in detail elsewhere (6–10). Finally, we have provided examples of the data that we have used to establish the optimal analysis settings used for routine analyses in our laboratory.

## Methods and materials

### Reagents

Dulbecco's sterile filtered replace with phosphate buffered saline (PBS) (Sigma, St. Louis, MO); Silica Microspheres, Colloidal (100, 150, 310 and 540 nm; Polysciences, Warrington, PA); Thermo Scientific Microsphere™ Polystyrene Size Standards [50, 60, 70, 100, 200, 300, 400 and 495 nm; National Institute of Standards and Technology (NIST) traceable; Thermo Fisher Scientific]; CellMask™ Orange plasma membrane stain (Invitrogen, Carlsbad, CA).

### Instrumentation

The data presented in this paper have been generated using 2 instruments: an NS500 equipped with a 488 nm laser, an EMCCD camera (Andor Technology, Japan) and zeta-potential measurement facility; and an NS500 equipped with a CMOS camera (Hamamatsu Photonics, Japan) with a 405 nm laser. The laboratory has previously worked with LM10, LM10-HS and LM14 (no longer available) devices. The same software release (NTA 2.3 build 013) was used throughout. Most of the software settings are proprietary and are not known to the authors.

### Capture settings

The camera gain and camera shutter speed may be set individually in *advanced* mode or an overall camera level may be set in *standard* mode. The camera gain ranges from 0 to 680 and the shutter speed ranges from 1 to 1,499 (equivalent to 0.47 to 50 ms). There are 16 camera levels ranging from the least sensitive (level 1: gain 0, shutter 1) to the most sensitive (level 16: gain 512, shutter 1,300).

### Analysis software settings

The following analysis settings are available with the NTA 2.3 software:

- *Detection threshold* determines the minimum intensity value of an image necessary for it to qualify as a particle to be tracked for analysis.
- *Minimum expected particle size* determines the maximum distance that the software will expect a particle to move from one frame to the next. This is dependent upon the particle size, i.e. a larger particle will move more slowly; therefore, the area searched by the software in the next frame will be smaller than that of a smaller fast moving particle. In NTA 2.3, this function may be automatically determined by the software.
- *Blur* defines the degree of smoothing designed to help eliminate noise, such as diffraction rings surrounding larger particles. An automatic blur setting can be used to apply an appropriate blur width based on the brightness of each pixel, causing brighter particles to be blurred more than dimmer particles. The automatic blur function is generally used.
- *Minimum track length* defines the minimum number of consecutive frames in which a particle must be visible before its size value is included in the size distribution plot. The size distribution profile produced by NTA is based only on the MSD of particles that have been tracked for a minimum number of frames (the minimum track length). With a high minimum track length, the particles are tracked for a greater length of time, resulting in more accurate sizing; however, the number of particles that have been tracked for the minimum number of frames (i.e. completed tracks) may be small. An automatic minimum track length is available and may be used in many circumstances.
- *Extract background*, as the name suggests, subtracts any pixels generated by background contamination from the image. This option should *always* be selected.

All particles that scatter enough light to be above the detection threshold are counted by the NTA software and this is used to determine their concentration. However, only those particles that are tracked for the minimum number of frames contribute to the size measurement. The size distribution graphs represent the particle size normalised to the particle count. If a sample is reasonably monodisperse and the measured concentration is optimal, the automatic minimum track length function may be used. If, however, the diluted sample has a low particle concentration or is highly polydisperse, better results may be achieved by using a lower minimum track length. This will increase the number of particles that are tracked but the sizing accuracy of individual particle sizes may be slightly reduced. *NB: Do not use a minimum track length of less than 5 as sizing accuracy will be compromised* (see ‘Results’).

### Instrument set up

#### Set up for LM10

The instrument should be sited away from obvious sources of vibration (e.g. centrifuges, freezers, etc.) and placed on a steady even surface; if this is not possible, an antivibration table may be necessary.

**Fig. 1:**
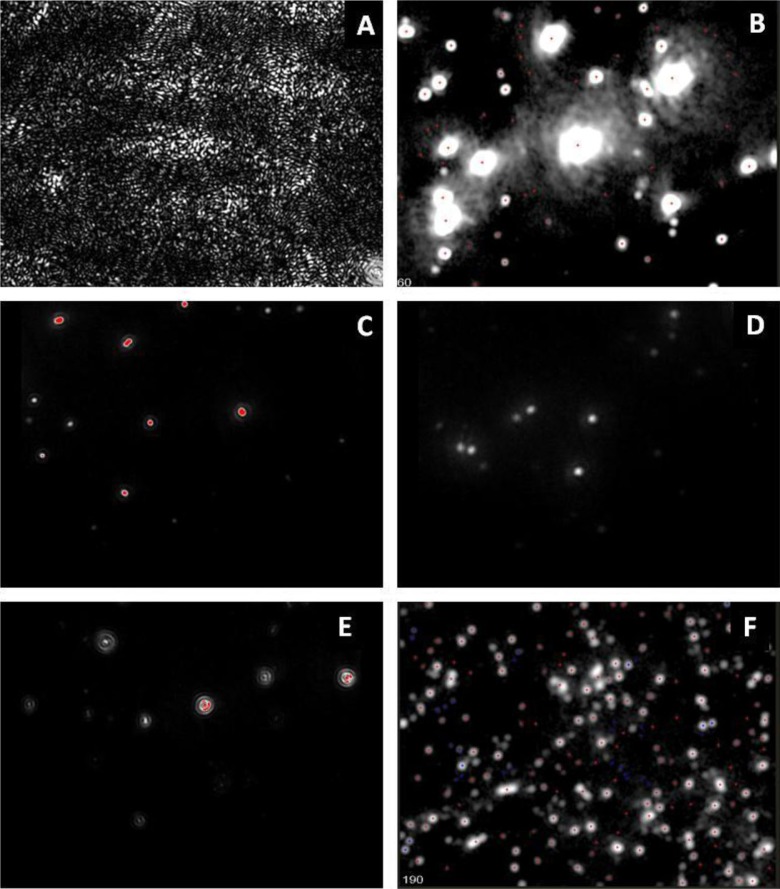
Onscreen images showing (A) the correct position of the “thumbprint” at the zero position; (B) overexposed particles due to inappropriately high camera settings; (C) a correctly focussed image of an appropriate concentration of particles; poorly focussed particles due to the stage being (D) too low or (E) too high; and a sample that is too concentrated for analysis.

Each instrument has a visual reference point on the surface of the optical flat with a “thumbprint-like shape”. Using the microscope stage adjustment knobs, locate the centre of the thumbprint (Fig. 1A).

The best imaging position is as close as possible to the thumbprint without obvious interference from light scattered by the thumbprint itself. In practice, this is usually between 1½ to 2 fields of view to the right of the thumbprint and the positioning is performed manually by the operator. It is important to use as close to the same position as possible for each analysis to minimize imprecision. Small deviations will have little effect, but at positions far from thumbprint, the laser is less well focussed, leading to reduced light intensity and a greater depth of illumination.

Introduce PBS into the chamber using a 1 ml syringe and check that it is free from particles and that the chamber is clean (i.e. no light scattering).

Select a silica microsphere of similar size to the EVs to be measured for calibration purposes (see the Standardisation section). As most EVs are polydisperse in size, a silica microsphere close to the modal size of the EVs is appropriate. The silica microspheres must be of known mass, density and mass/volume of diluent, so that the concentration per ml can be calculated (an Excel file used to calculate concentration may be downloaded from www.nanosight.com).

Empty the chamber and using a clean syringe, introduce the silica microsphere suspension into the sample chamber. After allowing a brief time to equilibrate (approximately 5–30 seconds), set the NTA mode to capture. Long delays between introducing the sample into the chamber and capture may lead to particles adhering to surfaces and inaccurate concentration measurements.

Gradually increase the camera level (which alters the shutter speed and gain) until the image is close to saturated (Fig. 1B), then slowly reduce the level until the microspheres are observed as single bright points (Fig. 1C), adjusting the focus if necessary. The microspheres should not be blurred (Fig. 1D) or produce diffraction rings (Fig. 1E). The manufacturer recommends that there should be approximately 20–60 microspheres per field of view, which approximates to a concentration in the region of 2 –10×108/ml. For some larger microspheres, it may be necessary to manually adjust the camera gain and shutter settings, but for most sizes, one of the fixed settings (1–16) should be adequate.

Capture a 30-second video, then introduce a fresh volume of sample into the chamber and make another recording. Repeat until 5 videos have been captured.

The chamber should be cleaned between samples and between different dilutions of the same sample by introducing clean PBS in order to avoid the risk of carryover, i.e. the presence of residual sample that could influence subsequent measurements. If residual particles are still visible, repeat the flushing process.

#### Set up for NS500

Select *go to zero* and check that the centre of the thumbprint (Fig. 1A) is visible. If not, the zero position may require adjustment.

Load the sample chamber slowly with PBS using the *prime fluidics* command.

Select the *scatter position*, which would be between 10,000 and 15,000 steps in from the zero position (indicated in the advanced stage communication window). There should be no visible particles and minimal light scattering.

The measurement procedure is essentially the same as for the LM10, but most functions may be automated. The use of automated settings reduces the degree of operator dependency and subjectivity.

Use the *load* function to introduce the silica microsphere suspension into the sample chamber and, after allowing a brief time to equilibrate (approximately 5 seconds), set the NTA mode to capture.

The adjustment of video capture settings is identical to that for the LM10. When visualisation of the silica microspheres is satisfactory, proceed to measurement.

In order to reduce variation due to operator technique and to prevent microsphere loss due to adhesion or settling, it is advisable to use the script control facility in the advanced tab to automate the analysis procedure. The following script is ideal for most purposes.

PUMPLOAD

DELAY 5

CAPTURE 30

PRIME

DELAY 5

CAPTURE 30

PRIME

DELAY 5

CAPTURE 30

PRIME

DELAY 5

CAPTURE 30

PRIME

DELAY 5

CAPTURE 30

This may be abbreviated as shown below.

PUMPLOAD

REPEATSTART

DELAY 5

CAPTURE 30

PRIME

REPEAT 5

The chamber should be flushed with PBS between samples. If residual particles are still visible, repeat the flushing process.

#### Video analysis

Ensure that the *extract background* box is ticked, then open the first recorded file and select auto for *detection threshold*, *blur*, *min track length* and *min particle size*.

Adjust the detection threshold using the ±function until a red cross is present in the centre of each particle. The presence of blue crosses indicates noise and shows that the threshold is too low, which will result in the measurement of false-positive events. The presence of particles without crosses indicates that the threshold is too high.

On the advanced menu, select *batch process*, select the appropriate batch of video files, and then select *go*.

Check that during the analysis, particle trajectories are generally turning from blue to red. Too many blue trajectory tracks that never turn red indicate that the minimum track length is set too high and that the particles are not being tracked to completion. Check that the 5 distribution curves are in agreement. If not, examine the videos for possible cause (presence of air bubbles, high background due to material sticking to the chamber) and take appropriate action (delete file or repeat measurement).

Mean size, modal size and concentration are displayed on the screen. The data summary files are (by default) automatically exported as comma separated value files (csv) that may be opened using Excel. If size distribution data is required, use the export multigraph set in the export tab.

### Standardisation

To date, most standardisation of NTA measurement has been performed using polystyrene microspheres (1, 2, 4, 5). These NIST-traceable polystyrene-size standards are ideal for verifying size measurements. However, polystyrene particles have a higher refractive index (approximately 1.59) than that reported for EVs (approximately 1.39) (11, 12). Using these reported refractive indices, Mie theory predicts that a polystyrene microsphere scatters approximately 4 times as much light as a vesicle of the same size. Silica microspheres, with a refractive index of 1.45, may be a more appropriate standard for concentration measurements made by NTA as they are thought to scatter a similar amount of light as EVs of similar size.

EV measurements made by NTA using settings determined by analysing polystyrene microspheres result in underestimation of EV numbers when compared to measurements made using settings established using silica microspheres (see the Results section).

The following protocol was used to establish the best capture and analysis settings for EV measurement by NTA.

Use data from several repeat measurements of microsphere dilutions of known concentration to assess whether the measured concentration is the same as the expected concentration and, if necessary, recalibrate using the following calibration factor:

$${\text{Calibration factor}=[\text{concentration}}_{\text{expected}}{/\text{concentration}}_{\text{measured}}]$$

It is advisable to check that the calibration factor is valid at several different concentrations across the intended measurement range to check that the relationship between expected and measured concentration remain linear. Subsequent EV measurements may then be multiplied by the calibration factor to obtain reproducible results.

*NB: The capture and analysis settings used to establish the calibration factor should be used for all subsequent EV measurements. Changing these settings may result in erroneous results*.

If different vesicle preparations with differing sizes are to be measured, it may be necessary to establish different settings using different-sized silica microspheres, e.g. 100 nm silica microspheres are appropriate when measuring exosome-sized vesicles of 80–130 nm, but larger microspheres may be necessary for vesicles released from activated cells or apoptotic cells, with a larger modal size. It is desirable to analyse at least 200–250 completed tracks per video (i.e. a minimum of 1,000 per sample).

*NB: This procedure should be performed whenever the software or hardware are changed or if the quality-control measurements are persistently out by more than 10%*.

### EV measurement procedure

At the start of each day or analysis session, the position of the thumbprint/analysis area should be checked to ensure that the measurements are being made in the correct region. A suspension of particles of known size and concentration (e.g. silica microspheres) should be analysed to check instrument performance (see the previous section). Ideally, the particles should be of a similar size to the vesicles normally analysed and the predicted concentration should be in the middle of the ideal measurement range, i.e. approximately 5×10^8^/ml. This should be performed on each day of use and the results recorded; a Levey-Jennings plot (a cumulative plot of measurements over time) can be useful for quality-control purposes. Most biological samples require dilution prior to NTA measurement. Ideally, the concentration should be 2–10×10^8^/ml, which is within the linear range (1). Above this concentration, the screen becomes crowded and it may be difficult to discern small EVs in a polydisperse sample. Analysing concentrations of EVs below 2×10^8^/ml results in reduced precision. As a general rule of thumb, the diluted sample should appear totally transparent. For measurements of EVs obtained by ultracentrifugation of blood plasma, a dilution factor of 10 to 20 (when compared to the original volume of plasma) typically yields good results. More concentrated suspensions may be necessary if the operator wishes to look specifically at the rarer large EVs. The optimum dilution for cell culture supernatants varies considerably.

The sample should be analysed within 15 minutes of the initial dilution to achieve acceptable levels of accuracy and precision. Introduce the diluted sample into the sample chamber. The volume required varies depending on the instrument model but is typically 0.3 to 0.6 ml. There should be no evident flow after the first few seconds. A delay of 5 seconds between sample introduction and the start of the first measurement is advisable.

Focus the image so the microspheres/vesicles appear as sharp points of light (Fig. 1B), without diffraction rings. Ideally, there should be approximately 20–60 particles per field of view. If the sample is too concentrated (Fig. 1F), it should be diluted further. For very low EV counts, it may be necessary to increase the number of videos captured in order to achieve an acceptable number of tracked events. If it is still not possible to obtain sufficient EVs to achieve a suitable concentration, it may be necessary to reduce the *minimum track length* to 10 or for very low EV counts reduce to 5. Ideally, at least 1,000 events in total should be tracked.

Analyse the videos using the *batch process* option in the advanced tab. Check that the analysis software settings are correct before pressing *GO*.

Check that the size distribution curves are in agreement, and in the export tab select *export multigraph* to send the size distribution data to Excel as a csv file.

### Fluorescence measurements

With the appropriate combination of laser wavelength, fluorophore labelled probe and long pass (which allow light above a defined wavelength to pass) or bandpass (which only allow light within a defined wavelength band) filter, it is possible to analyse fluorescently labelled EVs. This involves the detection of emitted fluorescent light, which has a longer wavelength than the absorbed light and is dependent on the Stokes shift of the fluorophore used. However, this is more involved than standard measurements and certainly requires more method development, as discussed below.

Due to the small size of EVs, it is frequently difficult to achieve the labelling efficiency required to produce the necessary signal to noise for detection of fluorescently labelled EVs. However, there are a number of tips that can help improve this. Careful titration of the probe and removal of unbound probe by washing (size-exclusion filter or high-speed centrifugation) are usually necessary for EV labelling. Photobleaching of fluorophores can be a significant issue for fluorescence NTA measurements. A synchronisation cable is fitted to all fluorescence instruments, which pulses the laser in time with the camera shutter to minimise the exposure of the fluorophore to the illumination source and reduce photobleaching. This may also be overcome by flowing the sample through the sample chamber using a slow pump speed on the NS500, or by using an integrated syringe pump (if fitted). It has also been found in several cases that increasing the antibody incubation times enhances fluorescence detection.

Quantum dot-conjugated antibodies have been used with some success (1). In our experience, this has no significant effect on vesicle size and we have seen no evidence of quantum dots binding multiple EVs. However, antibody/quantum dot conjugation can be difficult, with reduced antibody reactivity and non-specific aggregation, leading to false negative and false positive results, respectively. It is essential therefore to perform isotype controls and to check the success of the antibody conjugation by flow cytometry. Fc-conjugated antibodies give the best binding performance, but the conjugation process requires a large amount of antibody, specialist equipment and consequently tends to be expensive unless the quantum dot-antibody conjugate is commercially available. Unbound quantum dots can interfere with NTA analysis, so careful titration and washing the labelled EVs through a 300 kd centrifugal filter at low speed (<1,000 g) reduces this problem. This is preferable to high-speed centrifugation, which we have found can result in dissociation of the label. Analysing a dilute preparation of the quantum dot conjugate alone should be performed to detect the presence of any non-specific quantum dots aggregates. It is usually advisable to block Fc receptors on EVs prior to labelling as most microvesicles in blood will bind Fc fragments. This is less of a problem if using Fc-conjugated antibodies or Fab fragments. Intercalating nucleic acid dyes such as SYBR Green (Invitrogen) also show promise.

## Technical notes

### General issues

- The *minimum expected particle size* determines the maximum distance (in pixels) from the particle's position in a given frame that the software will search for a particle in the next frame. It also establishes an exclusion zone around a particle of the same radius, so that if another particle enters this exclusion zone, then the software excludes the information from both particles. If the *minimum expected particle size* is set too low, this can lead to erroneous results, as 2 or more larger particles may be tracked as a single small particle. The appearance of apparently small very bright particles in the intensity versus scatter plot should alert the operator to the likelihood that the *minimum particle size* has been set too low. Unless the operator has prior knowledge of the size of the particles in a sample (which is rarely the case in EV measurement), it is advisable to use the automatic setting.
- It is essential that all culture media and diluents are particle free. This should be verified prior to any measurement. Ultracentrifugation or filtration may be necessary to achieve this.
- If the sample to be studied is highly polydisperse, it may be necessary to analyse the sample at more than one concentration using different camera settings in order to accurately characterise all EVs. As the larger particles (>150 nm) are usually present in smaller numbers, the best results may be achieved using a suspension 10 times more concentrated than is usually required when analysing smaller particles, with capture settings obtained from 200 or 300 nm silica microspheres. The smaller EVs may then be analysed on a dilute suspension using capture settings obtained from 100 nm silica microspheres, as illustrated in the Results section. By using the negative gating function (performed by right-clicking the mouse in the size versus light intensity window) or using the cursors at the bottom of the main analysis window, it is possible to analyse EVs within a specified size range. *NB: It is important to appreciate that if very large numbers of large EVs are present, there will be an overestimation of small EVs due to multiple light-scattering events*.
- Concentration measurements decrease with time due to adherence to the sample chamber and tubing. This can be minimised by making several short measurements (e.g. 5×30 seconds) rather than one long measurement and by introducing a fresh sample into the chamber for each of the short measurements. With the NS500, this is best achieved by incorporating a PRIME step between each measurement in the script control rather than ADVANCE (which results in a gradual decrease in measured concentration). When using the LM10 instruments, this is done manually.
- Too much protein in the sample may cause optical noise during NTA measurements and lead to a build-up of protein within the sample chamber, resulting in an increase in background light scattering.
- Regular quality control measurements can help predict when a change in calibration factor or preventative maintenance is required. For example, hardware and software upgrades may affect the absolute measurements obtained. Deterioration of the condition of the optical flat or changes in the laser light path over time may also cause significant changes.

### Issues specific to the measurement of EVs

- The temperature for EV measurement should not exceed 37°C. Ambient temperature appears to be satisfactory for all measurements.
- Distilled water is not suitable for measuring EVs.
- In samples of limited size and few EVs, it may be necessary to perform a larger number of replicate measurements (e.g. 10×30 s). This is preferable to increasing the time of each video capture as fewer EVs are lost to adhesion. In the analysis of samples with low EV concentration, the cleanliness of the instrument and diluent are critical.
- If EVs are isolated by ultracentrifugation it is advisable where possible to resuspend the pellet in PBS containing 0.1 µm filtered 0.01% albumin to avoid EV loss during analysis due to adhesion. This should be checked for particles prior to use and may require further filtration or ultracentrifugation. Sodium azide may be added at a concentration of 0.1% as a preservative. The pellet produced by ultracentrifugation must be thoroughly resuspended by pipetting up and down. *NB: NTA cannot distinguish EV aggregates from large EVs*.
- When using blood plasma/serum it is always necessary to isolate the vesicles by ultracentrifugation to remove lipoprotein particles, as these are of a similar size to most EVs and are typically present at concentrations in excess of 1×10^12^/ml, thus hugely outnumbering the EVs.
- Samples prepared from sucrose gradient centrifugation need to be diluted at least 100-fold to attenuate the effect of sucrose on viscosity. If this is not possible or if the operator suspects that the viscosity of the suspension is higher than that of PBS, the viscosity should be measured and the appropriate value entered into the NTA software prior to video analysis.

## Troubleshooting

### Sample drift

Sample drift should not occur during normal use. Although the software can correct for sample drift (enable the *Auto Drift Correction* option in the *Advanced* tab), sample drift should not occur during normal operation and indicates a problem with the analyser. This can be caused by the presence of air bubble in the sample chamber, leaking diluent from the O-ring in the LM10, loose tubing connectors, poor pump/tubing contact, or worn tubing in the NS500. The use of inappropriately high levels of sucrose in samples prepared by sucrose density gradient separation can also cause drift due to osmotic pressure across the chamber.

### Incorrect concentration measurement for microspheres

The most common cause of incorrect concentration measurements is inaccurate pipetting, so it is always worth making another dilution before proceeding with the next steps. Silica microspheres can aggregate after prolonged storage, which can usually be rectified by a sonication but may indicate that the microspheres need replacing. High background light scattering can also cause inaccurate concentration measurements.

### Air bubbles

Air bubbles can deflect the laser, cause background scattering events and produce sample drift. Air bubbles may be removed by reintroducing the sample or rinsing the chamber with PBS and reintroducing the sample. However, if these measures do not work, it may be necessary to clean the optical flat and chamber.

### High background

There are several potential causes of a high background. A build-up of material on the optical flat can cause non-specific light scattering that may cause false positive events and may also lead to reduced sensitivity to smaller EVs. Flushing the system with PBS and/or distilled water may dislodge the material, but if this does not reduce the background, it is necessary to dismantle the sample chamber to clean the optical flat and chamber top plate. This is best achieved by washing with distilled water via a wash bottle and wiping the optical flat firmly with a tissue soaked in 70% alcohol or a dry tissue. *NB: The NS500 gasket is easily damaged and care must be taken during cleaning and drying*. If this does not reduce the background, the manufacturer/distributor should be consulted. If particles are visible in the chamber after flushing, this is most likely due to contamination of the rinse solution or deterioration of the tubing (NS500 model). It is good practice to regularly change the rinse solution and to clean the optical flat after heavy usage.

### Incorrect size measurement of microspheres

An unexpected low diameter may be caused by vibration, using the wrong viscosity, or temperature measurement. Falsely high size measurements may be caused by aggregation of the microspheres (check measurement with another preparation), using the wrong viscosity value or temperature value. If the problem persists after eliminating these possible causes, refer the problem to the manufacturer/distributor.

## Results

### The use of low refractive index microspheres for concentration standards

It has been reported that EVs have a refractive index much lower than that of polystyrene microspheres and that silica microspheres may be a more suitable material for standardising EV measurements (11). In order to test this, we measured the relative intensity of light scattered by 100 nm polystyrene and 100 nm silica microspheres. We then measured a suspension of EVs prepared from human plasma and recorded the light-scattering intensity for those EVs with a measured diameter of between 90 and 110 nm. The amount of light scattered by EVs, silica and polystyrene microspheres was in the ratio of 1: 1.5: 2.57 for EVs, silica and polystyrene, respectively. Using the known refractive indices of silica (1.45) and polystyrene (1.59), and applying the Rayleigh approximation, an estimated refractive index for plasma EVs of 1.41 was obtained (see the supplementary data). Assuming this value to be correct, the measured scattered light intensity agrees well with the scattering ratio of 1: 1.5: 3.16 predicted by the Rayleigh approximation. However, it cannot be excluded that the refractive index may substantially differ between vesicles of different origins.

In order to study the importance of these findings, silica microspheres of 100 nm and 540 nm were used to establish ideal acquisition settings for the quantitation of small and large low refractive index particles. Known concentrations of 100 nm and 540 nm silica microspheres and 100 nm and 485 nm polystyrene microspheres were analysed using these settings. The procedure was then reversed, so that acquisition settings were established using polystyrene microspheres and the same microspheres were then reanalysed. As can be seen from Table I, the calibration using polystyrene microspheres leads to an underestimation of silica microspheres and vice versa. Using settings established using 60 nm microspheres, it is not possible to resolve silica microspheres of the same size (data not shown), which suggests that silica is a better material for estimating the lowest detection threshold of the system for EVs.

**Table I:** The effect of low (silica) and high (polystyrene) refractive index microspheres on establishing the optimum calibration settings for concentration measurements

Microsphere type	Expected concentration	Measured concentration
Polystyrene microsphere settings		
Silica 100 nm	5.21	1.84 (0.48)
Polystyrene 100 nm	4.84	5.14 (0.21)
Silica microsphere settings		
Silica 100 nm	5.21	5.26 (0.35)
Polystyrene 100 nm	4.84	10.94 (0.57)
Polystyrene microsphere settings		
Silica 540 nm	3.13	0.46 (0.38)
Polystyrene 485 nm	2.99	3.02 (0.31)
Silica microsphere settings		
Silica 540 nm	3.13	3.36 (0.39)
Polystyrene 485 nm	2.99	7.33 (0.61)

Measured concentrations represent the mean (and standard deviation) of 5 measurements. These experiments were performed using an NS500 equipped with a 488 nm laser and an EMCCD camera. The following settings were used: camera level 10 for 100 nm silica microspheres; camera level 8 for polystyrene microspheres; camera shutter speed 25 and gain 10 (between level 3 and level 4) for 540 nm silica microspheres; and camera level 2 for 485 nm polystyrene microspheres.

Using an NS500 instrument equipped with a 405 laser and a CMOS camera, it is possible to obtain accurate measurements of 60 nm silica microspheres. However, the limit of resolution varies between different instruments and should be established for each instrument.

### The effect of minimum track length

**Fig. 2:**
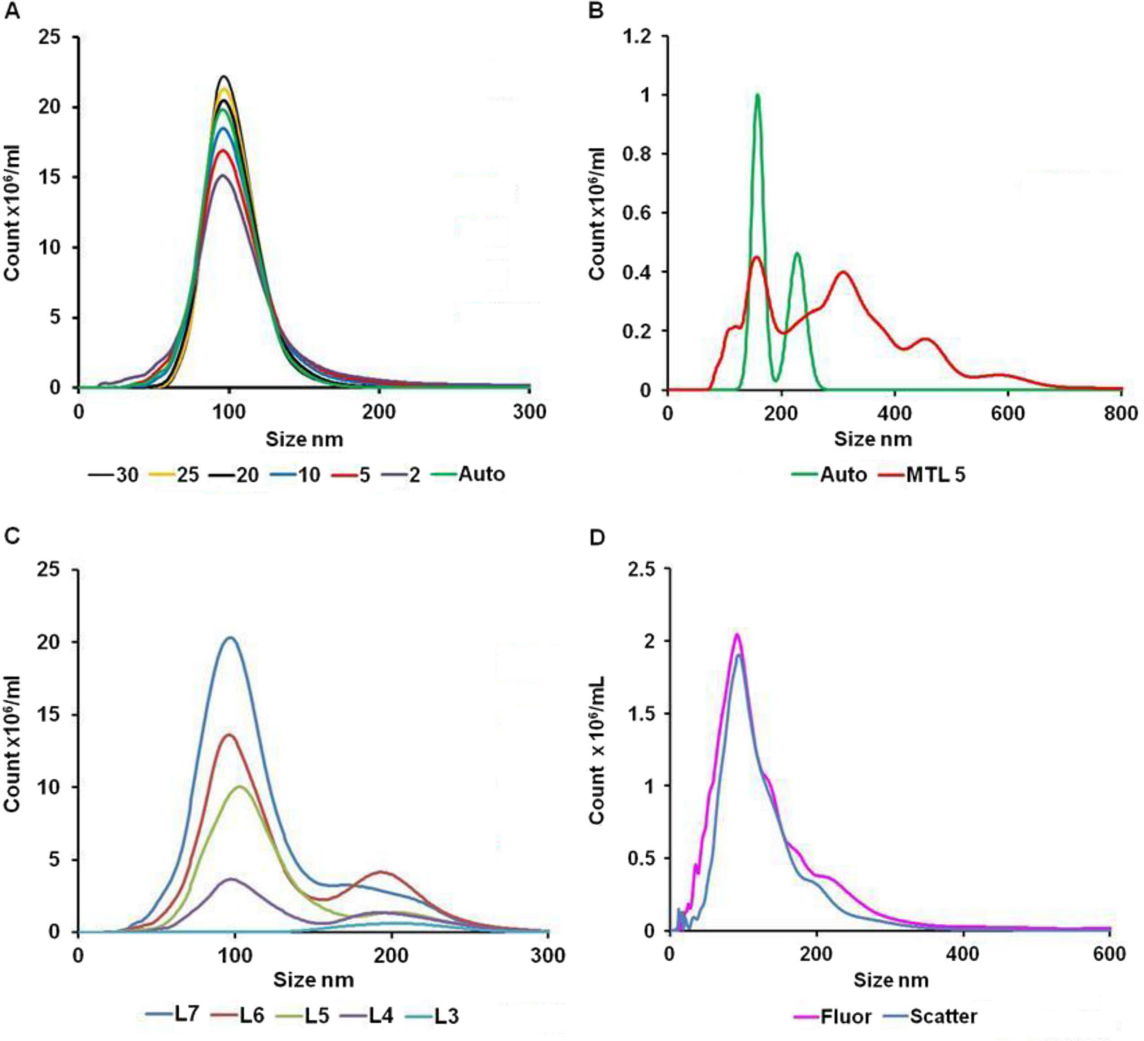
(A) The effect of minimum track length (MTL) on measured size distribution of monodisperse 100 nm silica microspheres; (B) the effect of using automatic (Auto) or manual (MTL5) minimum track length on measurement of a low concentration of polydisperse EVs; (C) the effect of increasing camera level (level 3 to level 7) on the measurement of a mixture of 100 nm and 200 nm microspheres (concentration 10×10^8^/ml and 0.5×10^8^/ml, respectively); and (D) NTA analysis of plasma EVs labelled with CellMask using light scattering (Scatter) and fluorescence (Fluor) measurement.

The size distribution profile produced by NTA is based only on the MRD of particles that have been tracked for a minimum number of frames (the minimum track length). While the automated function is suitable for many situations, only a minority of particles (approximately 20%) are sized, which is acceptable when a large number of EVs are tracked but may not be suitable if the diluted sample has a low EV concentration or the sample is polydisperse. In order to study the effect of minimum track length on sizing accuracy and the percentage of particles tracked, 100 nm silica microspheres were run and the videos analysed using the a range of minimum track lengths from 2 to 25 in addition to the automated setting (Fig. 2A). At a minimum track length of 5, 40–50% of all particles are tracked and acceptable sizing accuracy is obtained, but at minimum track lengths of less than 5, sizing accuracy is severely compromised (Table II). *NB: The minimum track length has no effect on concentration measurements but determines the proportion of events used to build the size distribution. However, an inappropriately low threshold value could lead to detection of false positive events or an overestimation of concentration*.

**Table II:** The effect of minimum track length on sizing accuracy of 100 nm microspheres

Minimum track length	Mean size (SD) nm	CV	completed tracks%
Automatic	101 (22)	22	19
25	103 (22)	21	11
20	104 (20)	19	15
18	103 (23)	22	16
16	104 (21)	20	18
14	105 (22)	21	20
12	105 (24)	23	23
10	106 (24)	23	26
9	106 (24)	23	28
8	106 (28)	26	32
7	107 (27)	25	35
6	107 (33)	31	39
5	108 (34)	31	45
4	109 (44)	40	52
3	115 (82)	71	71
2	124 (119)	96	100

When samples containing very low EV numbers are analysed using the automated minimum track length, this can result in unrepresentative size distribution profiles based on the completed tracks of a small number of EVs. In this situation, it is appropriate to reduce the minimum track length to 5 as a more representative size distribution generated from a larger number of tracks is obtained, as demonstrated in Fig. 2B.

#### Dealing with polydisperse samples

As with any light-scattering technique, the concentration of larger particles tends to be overestimated, as they scatter far more light. 100 nm and 200 nm microspheres were mixed at final concentrations of 10×10^8^/ml and 0.5×10^8^/ml, respectively. Figure 2C shows how the observed concentration of the 2 populations changes with camera level. At level 3, no 100 nm microspheres are observed, but 0.52×10^8^/ml 200 nm microspheres are measured. At level 7, a total of 13.40×10^8^/ml microspheres are observed of which 10.89×10^8^/ml are below 150 nm in size. Thus, by using the cursors to define populations of certain size, it is possible to obtain estimations of different-sized particles at the appropriate settings; in this case, level 3 for 200 nm microspheres and level 7 for 100 nm microspheres. In polydisperse samples in which there are many large EVs, it is difficult to accurately assess the number of small EVs as they are partially obscured by the larger brighter EVs. Fortunately, in our experience, the number of small EVs usually far exceeds the larger EVs, so that the large EVs may be diluted out and the smaller EVs analysed. Measuring samples with a high degree of polydispersity remains a significant challenge and more work needs to be done to improve the performance of NTA in this setting.

#### EV measurements

Human platelet free plasma from normal individuals typically contains 1–5×10^12^ particles per ml but over 98% of these are not pelleted by ultracentrifugation and appear to be lipoprotein particles. Ultracentrifugation isolates 0.5–5.0×10^10^ EV/mL with a modal size of 70–120 nm. We have verified that these EVs are membrane derived through the use of membrane-penetrating peptides conjugated to quantum dots (1) and using the amphoteric fluorescent dye, CellMask Orange plasma membrane stain (Fig. 2D). This concentration value is many times higher than that obtained by conventional count flow cytometers (7).

Exosomes from cell culture supernatants typically have a modal size of 90–160 nm (13–16). This is larger than the values normally quoted in the literature, which have been obtained by electron microscopy of fixed dehydrated samples and may have suffered shrinkage during preparation as illustrated by the observed “cup shaped” morphology, which is probably an artefact. EVs derived from activated or apoptotic cells tend to be larger but vary greatly in size (17, 18).

Depending on the characteristics of the instrument used (camera type, laser wavelength, etc.), EVs of <90 nm in diameter may not be resolved, resulting in an overestimation of EV size. Furthermore, as most EV preparations are polydisperse in nature, it may be necessary to perform more than one measurement using different camera levels to obtain satisfactory results.

## Discussion

Here we present a protocol for the standardisation of measurement of EVs by NTA. We have used silica microspheres to standardise these measurements as the refractive index of silica is similar, but not identical, to that of EVs. Clearly, a biological standard is highly desirable, but this presents several problems: which method would be used to standardise the standard?; the standard would have to be stable at 4°C or withstand freezing and thawing with no change in size or concentration for months or even years; ideally, the biological standard would be detectable by fluorescence; and it would be available in large amounts with predictable qualities. Work is currently under way to produce biological standards suitable for EV measurement by many different techniques, but this may take many years. Until this time, calibration with silica microspheres appears to offer the best compromise.
